# Isolated low levels of high‐density lipoprotein cholesterol and stroke incidence: JMS Cohort Study

**DOI:** 10.1002/jcla.23087

**Published:** 2019-11-19

**Authors:** Jun Watanabe, Eiichi Kakehi, Kazuhiko Kotani, Kazunori Kayaba, Yosikazu Nakamura, Shizukiyo Ishikawa

**Affiliations:** ^1^ Center for Community Medicine Jichi Medical University Shimotsuke Japan; ^2^ Department of General Medicine Tottori Municipal Hospital Tottori Japan; ^3^ Graduate School of Saitama Prefectural University Koshigaya Japan

**Keywords:** cohort studies, high‐density lipoprotein, incidence, Japanese, stroke

## Abstract

**Background:**

The cardiovascular relevance of isolated low levels of high‐density lipoprotein cholesterol (HDL‐C) is yet to be determined. Stroke often leads to long‐term disability, and thus, not only stroke mortality but also stroke incidence is a topic of research. Although isolated low HDL‐C level has been found to be a predictor for stroke mortality previously, whether it can predict stroke incidence is unknown.

**Methods:**

In the Jichi Medical School cohort study, 11 025 community‐living residents without a history of stroke were examined. Hazard ratios (HRs) for isolated and non‐isolated low HDL‐C levels were calculated relative to those for normal HDL‐C levels in stroke patients using Cox's regression models.

**Results:**

During the mean follow‐up period of 10.7 years, 412 residents had their first‐ever stroke. The multivariable‐adjusted HRs for the levels of isolated and non‐isolated low HDL‐C were 1.11 (95% confidence interval, 0.85‐1.44) and 1.35 (1.01‐1.81), respectively, when compared to that for normal HDL‐C.

**Conclusion:**

Low HDL‐C levels with other dyslipidemias may contribute to the incidence of stroke, not isolated low HDL‐C.

## 1 INTRODUCTION

Stroke is the most common cause of disability and mortality and is a concerning health issue worldwide.^1^ Although there has been a marked reduction in stroke‐related mortality, its burden has been increasing for decades.^2^ Research not only on stroke‐related mortality but also its incidence is necessary to understand the recent trends in stroke.

Low levels of high‐density lipoprotein cholesterol (HDL‐C) are reported to be associated with an increased risk of stroke.^3^, ^4^, ^5^, ^6^, ^7^, ^8^ However, most studies did not adjusted other lipid abnormalities. The relevance of isolated low levels of HDL‐C in stroke remains undetermined. Previous studies have reported that the levels of isolated low HDL‐C do not predict stroke‐related mortality,^9^, ^10^ but whether it can predict the incidence of stroke remains unknown. Approximately 90% of patients who experience a first‐ever stroke survive the first month.^11^ Factors associated with the incidence and mortality of stroke can be different. Herein, the predictive relationship of isolated low HDL‐C levels with the incidence of first‐ever stroke was investigated among general Japanese residents in a cohort study.

## 2 MATERIALS AND METHODS

The study was a serial analysis of the Jichi Medical School (JMS) Cohort Study, a multiregional community‐based prospective cohort study with baseline data collected between April 1992 and July 1995 from national population screening tests according to the Health and Medical Service Law for the Aged in Japan that assessed the incidence of stroke until the end of 2005.^12^ Among 12 490 participants (4911 men and 7579 women), we excluded the following 1465 participants: 102 without follow‐up data, 112 with a history of stroke, 1146 without data on the stroke history, and 105 with no data of HDL‐C levels, triglyceride, and total cholesterol. A total of 11 025 residents (4283 men and 6742 women) aged 18‐90 years without a history of stroke were analyzed. This study was approved by the Institutional Review Board of Jichi Medical University (No.06‐11, 2006), and all the participants provided written informed consent.

As previously described,^12^ body mass index (BMI) was calculated from the resident's height and weight. The systolic and diastolic blood pressure (BP) levels were measured twice using a sphygmomanometer and appropriate cuff size in a sitting position after resting for 5 minutes. The serum levels of total cholesterol (TC), triglyceride (TG), HDL‐C, fasting and postprandial plasma glucose (PG), and creatinine (Cr) were measured enzymatically (SRL Inc, Tokyo, Japan). The questions related to lifestyle by trained interviewers included: smoking status (never, past, or current), alcohol drinking status (never, past, or current), daily alcohol consumption, medication history (current use of antihypertensive, antidiabetic, and antihyperlipidemic medications), and physical activity (the Framingham Study Questionnaire).^13^ Low‐density lipoprotein (LDL) was calculated using the Friedewald formula where LDL‐C = TC – HDL‐C + (TG/2.2).^14^ Estimated glomerular filtration rate (eGFR) was calculated using the Chronic Kidney Disease Epidemiology Collaboration equations modified by a Japanese coefficient: male, Cr ≤ 0.9 mg/dL, 141 × (Cr/0.9)^−0.411^ × 0.993^age^ × 0.813; Cr > 0.9 mg/dL, 141 × (Cr/0.9)^ −1.209^ × 0.993^age^ × 0.813; female, Cr ≤ 0.7 mg/dL, 144 × (Cr/0.7)^ −0.329^ × 0.993^age^ × 0.813; Cr > 0.7 mg/dL, 141 × (Cr/0.7)^−1.209^ × 0.993^age^ × 0.813.^15^ We classified HDL‐C into 3 categories: (1) isolated low HDL‐C (HDL‐C < 1.0 mmol/L with TG < 1.7 mmol/L and TC < 6.2 mmol/L); (2) non‐isolated low HDL‐C (HDL‐C < 1.0 mmol/L with TG ≥ 1.7 mmol/L and/or TC ≥ 6.2 mmol/L); and (3) normal HDL‐C (HDL‐C ≥ 1.0 mmol/L), based on the National Cholesterol Education Program Adult Treatment Panel III (NCEP‐ATP III).^10^

The participants were interviewed face‐to‐face to confirm whether they had experienced their first stroke through annual follow‐up surveys. Participants without complete follow‐up examinations were contacted by mail or telephone. The medical histories of the participants were also confirmed if they had been treated in a hospital. We annually obtained death certificates from the public health centers with the permission of the Agency of General Affairs and the Ministry of Health, Labor and Welfare. The diagnosis committee consisted of a radiologist, a neurologist, and 2 cardiologists who independently diagnosed the stroke subtypes using the criteria by the National Institute of Neurological Disorders and Stroke.^16^

The continuous variables were expressed as mean and standard deviation or median and interquartile range and categorical variables were expressed as numbers and percentages. The continuous variables included age, BMI, SBP, DBP, PG, TC, TG, HDL‐C, LDL‐C, and eGFR, and categorical variables included sex, smoking status, alcohol drinking, antihypertensive, antihyperlipidemic or antidiabetic medication, and physical activity. The comparisons between the groups were performed using a one‐way analysis of variance or the chi‐squared tests. In the present study, a complete‐case analysis was used. A multivariable analysis was performed using the Cox's proportional hazards model to calculate the adjusted hazard ratios (HRs) and their 95% confidence intervals (CIs) for stroke incidence with isolated low HDL‐C or non‐isolated low HDL‐C, using normal HDL‐C as reference. Model 1 was adjusted for age and sex. Model 2 was adjusted for the variables in Model 1 plus BMI, systolic BP, smoking status, alcohol drinking, physical activity, plasma glucose, and antihypertensive, antihyperlipidemic or antidiabetic medication. A value of <0.05 was set as the significance level. All statistical analyses were performed with IBM SPSS version 25.0 (IBM Corp., Armonk, NY, USA). Data of participants under study were safely stored in a locked safe and in such a way that individuals are unidentifiable and analyzed on the encrypted computer without internet connection.

## 3 RESULTS

Table 1 shows the baseline characteristics of the participants. The mean HDL‐C level was 1.33 mmol/L. Only 2.6% of participants had HDL‐C levels ≥ 2.07 mmol/L. A total of 412 strokes were confirmed during the mean follow‐up period of 10.7 years.

**Table 1:** Baseline characteristics of participants according to categories of HDL‐C^a^

Categories	Normal HDL‐C		Isolated low HDL‐C		Low HDL‐C and higher TC and/or TG		*P* b
All participants	N		N		N		
Age (y)	9053	55.0 (11.5)	1020	56.2 (12.2)	952	55.1 (11.0)	.008
Men, %	3226	35.6	538	52.7	519	54.5	<.001
BMI (kg/m^2^)	8919	22.8 (3.0)	1001	23.6 (3.1)	939	24.9 (2.9)	<.001
SBP (mmHg)	8982	128.8 (20.9)	1011	128.4 (20.5)	944	133.2 (20.3)	<.001
DBP (mmHg)	8982	77.1 (12.3)	1011	76.4 (12.0)	944	79.9 (12.3)	<.001
Plasma Glucose (mmol/L)	9039	5.27 (4.88‐6.00)	1019	5.27 (4.88‐5.88)	952	5.66 (5.05‐6.55)	<.001
Fasting plasma glucose, %	4174	46.1	532	52.2	358	37.6	<.001
Total cholesterol (mmol/L)	9053	5.01 (0.89)	1020	4.39 (0.78)	952	5.18 (0.94)	<.001
Triglyceride (mmol/L)^a^	9053	1.00 (0.73‐1.40)	1020	1.17 (0.91‐1.40)	952	2.39 (1.98‐3.23)	<.001
HDL‐cholesterol (mmol/L)	9053	1.42 (0.62)	1020	0.90 (0.10)	519	0.86 (0.11)	<.001
LDL‐cholesterol (mmol/L)	9053	3.05 (0.82)	1020	2.97 (0.74)	952	3.04 (1.03)	.006
eGFR (mL/min/1.73m^2^)	4576	82.1 (12.3)	268	61.3 (13.5)	28	66.8 (15.6)	<.001
Current smoking, %	1920	21.2	323	31.7	296	31.1	<.001
Current alcohol drinking, %	3954	43.7	417	40.9	400	42.0	.379
Alcohol drinking, g/day	8457	16.9 (29.7)	946	14.1 (24.1)	885	18.3 (37.9)	.007
Diabetes Mellitus, %	155	1.7	26	2.5	30	3.2	.003
Hypertension, %	955	10.5	104	10.2	120	12.6	.128
Hyperlipidemia, %	147	1.6	12	1.2	27	2.8	.009
Physical activity, PAI ≥ 40, %	933	10.3	123	12.1	103	10.8	.605

Abbreviations: BMI, body mass index; DBP, diastolic blood pressure; HDL‐C, high‐density lipoprotein cholesterol; PAI, physical activity index; SBP, systolic blood pressure.

a Data are shown as mean (standard deviation) or median (interquartile range: plasma glucose and triglyceride) for quantitative data and as percentage of participants for qualitative data.

b *P* levels were calculated using one‐way analysis of variance or chi‐square test.

Appendix S1 shows multivariable‐adjusted HRs and 95% CI for incident stroke and stroke subtypes according to the three categories of HDL‐C. Isolated low HDL‐C was not found to predict incident stroke (Model 1: HR, 1.02; 95% CI, 0.79‐1.31; Model 2:1.11, 0.85‐1.44). The participants with non‐isolated low HDL‐C were found to have a higher risk for incident stroke as compared to those with normal HDL‐C (Model 1: HR, 1.44, 95%CI: 1.10‐1.88; Model 2:1.35, 1.01‐1.81). No significant associations were found between isolated low HDL‐C and stroke subtypes (subarachnoid hemorrhage, intracerebral hemorrhage, and cerebral infarction; data not shown) or between isolated low HDL‐C and infarction subtypes (lacunar infarction and atherothrombotic cerebral infarction; data not shown).

## 4 DISCUSSION

This JMS cohort study demonstrated that isolated low HDL‐C was not a predictor for the incidence of stroke in the general Japanese population, although low HDL‐C with other dyslipidemias (higher TC and/or TG) was found to predict stroke. The results of this study on stroke incidence appeared to be similar to those of previous studies on stroke mortality (meta‐analysis in the Asia‐Pacific Region^9^ and EPOCH‐JAPAN^10^), which reported that isolated low HDL‐C was not significantly associated with stroke mortality in men and women combined. As the cardiovascular relevance of isolated low HDL‐C is not determined, the absence of a relationship between isolated low HDL‐C and stroke incidence and mortality would add to the pathophysiological knowledge on isolated low HDL‐C.

Of note, the previous studies reported that the non‐isolated low HDL‐C did not predict the stroke mortality but predicted cerebral hemorrhage.^9^, ^10^ In our study, the non‐isolated low HDL‐C could predict the incidence of stroke. The difference in the outcomes for stroke may produce the predictability of non‐isolated low HDL‐C as different factors associated with the incidence and mortality of stroke are assumed.^11^ Although the true reason is unknown, it is hypothesized that lipids are carefully managed after a stroke, which leads to the lack of a relationship between non‐isolated low HDL‐C and stroke mortality.^17^ The observation that adding dyslipidemias to low HDL‐C other than simple low HDL‐C can contribute to incident stroke may be useful to prevent stroke, as dyslipidemias other than HDL‐C can be modulated by drugs.

This study has several limitations. First, chronic disease affecting HDL‐C, such as liver cirrhosis, nephrotic syndrome, and steroid‐treated disorders, could not completely be excluded, although these patients were less likely to participate in general checkups in the communities where the study subjects were recruited in our study. Second, HDL‐C level was measured once at baseline, and no assessment of changes in HDL‐C during follow‐up was undertaken. However, this approach is consistent using that applied in previous many prospective cohort studies.

In conclusion, our study suggested that isolated low HDL‐C might not predict stroke incidence, but non‐isolated low HDL‐C could predict stroke incidence. These findings would add to the knowledge on the role of low HDL‐C in stroke prevention.

## CONFLICT OF INTEREST

The authors disclose no conflicts of interest.

## Data Availability

The datasets analyzed in this study are available from the corresponding author (S. Ishikawa, i-shizu@jichi.ac.jp) on reasonable request.

## References

[1] Wang H , Naghavi M , Allen C , et al. Global, regional, and national life expectancy, all‐cause mortality, and cause‐specific mortality for 249 causes of death, 1980–2015: a systematic analysis for the Global Burden of Disease Study 2015. Lancet. 2016;388(10053):1459‐1544. 27733281 10.1016/S0140-6736(16)31012-1 PMC5388903

[2] Feigin VL , Nichols E , Alam T , et al. Global, regional, and national burden of neurological disorders, 1990–2016: A systematic analysis for the Global Burden of Disease Study 2016. Lancet Neurol. 2019;18(5):459‐480. 30879893 10.1016/S1474-4422(18)30499-X PMC6459001

[3] Wannamethee SG , Shaper AG , Ebrahim S . HDL‐Cholesterol, total cholesterol, and the risk of stroke in middle‐aged British men. Stroke. 2000;31:1882‐1888. 10926951 10.1161/01.str.31.8.1882

[4] Soyama Y , Miura K , Morikawa Y , et al. High‐density lipoprotein cholesterol and risk of stroke in Japanese men and women: The Oyabe Study. Stroke. 2003;34:863‐868. 12637692 10.1161/01.STR.0000060869.34009.38

[5] Zhang Y , Tuomilehto J , Jousilahti P , Wang Y , Antikainen R , Hu G . Total and high‐density lipoprotein cholesterol and stroke risk. Stroke. 2012;43:1768‐1774. 22496337 10.1161/STROKEAHA.111.646778

[6] Chei CL , Yamagishi K , Kitamura A , et al. High‐density lipoprotein subclasses and risk of stroke and its subtypes in Japanese population: The Circulatory Risk in Communities Study. Stroke. 2013;44:327‐333. 23321451 10.1161/STROKEAHA.112.674812

[7] Nakamura H , Mizuno K . Cardiovascular and cancer events in hyper‐high‐density lipoprotein cholesterolemic patients: A post hoc analysis of the MEGA study. Lipids Health Dis. 2014;13:133. 25135178 10.1186/1476-511X-13-133 PMC4246450

[8] Saito I , Yamagishi K , Kokubo Y , et al. Association of high‐density lipoprotein cholesterol concentration with different types of stroke and coronary heart disease: The Japan Public Health Center‐based prospective (JPHC) study. Atherosclerosis. 2017;265:147‐154. 28888808 10.1016/j.atherosclerosis.2017.08.032

[9] Huxley RR , Barzi F , Lam TH , et al. Isolated low levels of high‐density lipoprotein cholesterol are associated with an increased risk of coronary heart disease: An individual participant data meta‐analysis of 23 studies in the Asia‐Pacific region. Circulation. 2011;124:2056‐2064. 21986289 10.1161/CIRCULATIONAHA.111.028373

[10] Hirata T , Sugiyama D , Nagasawa SY , et al. A pooled analysis of the association of isolated low levels of high‐density lipoprotein cholesterol with cardiovascular mortality in Japan. Eur J Epidemiol. 2017;32:547‐557. 27709448 10.1007/s10654-016-0203-1

[11] Takashima N , Arima H , Kita Y , et al. management and short‐term outcome of stroke in a general population of 1.4 million japanese–Shiga Stroke Registry. Circ J. 2017;81:1636‐1646. 28579600 10.1253/circj.CJ-17-0177

[12] Ishikawa S , Gotoh T , Nago N , Kayaba K . The Jichi Medical School (JMS) Cohort Study: Design, Baseline Data and Standardized Mortality Ratios. J Epidemiol. 2002;12(6):408‐417. 12462275 10.2188/jea.12.408 PMC10681813

[13] Kannel WB , Sorlie P . Some health benefits of physical activity. Arch Intern Med. 1979;139:857‐861. 464698

[14] Friedewald WT , Levy RI , Fredrickson DS . Estimation of the concentration of low‐density lipoprotein cholesterol in plasma, without use of the preparative ultracentrifuge. Clin Chem. 1972;18:499‐502. 4337382

[15] Horio M , Imai E , Yasuda Y , Watanabe T , Matsuo S . Modification of the CKD epidemiology collaboration (CKD‐EPI) equation for Japanese: accuracy and use for population estimates. Am J Kidney Dis. 2010;56:32‐38. 20416999 10.1053/j.ajkd.2010.02.344

[16] Adams HP Jr , Bendixen BH , Kappelle LJ , et al. Classification of subtype of acute ischemic stroke. Definitions for use in a multicenter clinical trial. TOAST. Trial of Org 10172 in Acute Stroke Treatment. Stroke. 1993;24:35‐41. 7678184 10.1161/01.str.24.1.35

[17] Edwards JD , Kapral MK , Fang J , Swartz RH . Trends in long‐term mortality and morbidity in patients with no early complications after stroke and transient ischemic attack. J Stroke Cerebrovasc Dis. 2017;26:1641‐1645. 28506592 10.1016/j.jstrokecerebrovasdis.2016.09.038

