# Supplementary material for: Isolated low levels of high‐density lipoprotein cholesterol and stroke incidence: JMS Cohort Study
Source: J Clin Lab Anal. 2019 Nov 19;34(3):e23087. doi: 10.1002/jcla.23087 (PMC7083490; doi:10.1002/jcla.23087)
Supplement: Supplementary file 1 [file JCLA-34-e23087-s001.docx]

**Supplement 1.** Multivariable adjusted hazard ratios and 95% confidence intervals for incident stroke and stroke subtypes according to the three categories of high-density lipoprotein cholesterol.

|  | | Normal HDL-C | Isolated low HDL-C | Low HDL-C and  higher TC and/or TG |
| --- | --- | --- | --- | --- |
| Stroke | Number of participants | 7,072 | 2,447 | 1,506 |
|  | Number of events | 256 | 85 | 71 |
|  | Parson-years | 75,743 | 69,545 | 16,150 |
|  | Incidence rate^a^ | 3.38 | 3.20 | 4.40 |
|  | Model 1^b^ | 1.00 | 1.02 (0.79–1.31) | **1.44 (1.10–1.88)** |
|  | Model 2^c^ | 1.00 | 1.11 (0.85–1.44) | **1.35 (1.01–1.81)** |
| Subarachnoid hemorrhage | Number of events | 29 | 38 | 11 |
|  | Incidence rate^a^ | 0.38 | 1.43 | 0.68 |
|  | Model 1^b^ | 1.00 | 0.94 (0.46–1.89) | 1.59 (0.79–3.20) |
|  | Model 2^c^ | 1.00 | 0.97 (0.48–1.97) | 1.57 (0.74–3.33) |
| Intracerebral hemorrhage | Number of events | 61 | 12 | 22 |
|  | Incidence rate^a^ | 0.81 | 0.45 | 1.36 |
|  | Model 1^b^ | 1.00 | 0.60 (0.32–1.11) | **1.83 (1.11–3.00)** |
|  | Model 2^c^ | 1.00 | 0.71 (0.37–1.33) | 1.61 (0.91–2.83) |
| Cerebral infarction | Number of events | 166 | 62 | 38 |
|  | Incidence rate^a^ | 2.19 | 2.34 | 2.35 |
|  | Model 1^b^ | 1.00 | 1.20 (0.89–1.61) | 1.27 (0.89–1.81) |
|  | Model 2^c^ | 1.00 | 1.26 (0.92–1.73) | 1.22 (0.83–1.80) |

HDL-C, high-density lipoprotein cholesterol; HR, hazard ratio

^a^ Incidence rate was expressed as per 1,000 person-years

^b^ Model 1 was adjusted for age, and sex (men or women).

^c^ Model 2 was adjusted for age, sex (men or women), body mass index, systolic blood pressure, smoking status (current, past, or never smoker), alcohol drinking status (current, past, or never drinker), plasma glucose, and use of antihypertensive, antihyperlipidemic, or antidiabetic medication (yes or no), physical activity (low, middle, or high).
